# Behavior of Elastoplastic Auxetic Microstructural Arrays

**DOI:** 10.3390/ma6030726

**Published:** 2013-02-28

**Authors:** Rivka Gilat, Jacob Aboudi

**Affiliations:** 1 Department of Civil Engineering, Faculty of Engineering, Ariel University Center, Ariel 44837, Israel; 2 Faculty of Engineering, Tel Aviv University, Ramat Aviv 69978, Israel; E-Mail: aboudi@eng.tau.ac.il

**Keywords:** negative Poisson ratio, homogenization, microstructural architectures, composite

## Abstract

A continuum-based micromechanical model is employed for the prediction of the elasto-plastic behavior of periodic microstructural arrays that can generate negative values of Poisson’s ratios. The combined effects of the negative Poisson’s ratio generated by the array microstructure and the elastoplastic behavior of the constituents are studied. A design methodology for the determination of the constituents’ properties of two-phase arrays that generate required values of negative Poisson’s ratio is considered.

## 1. Introduction

Foam structures with negative Poisson’s ratio have been produced by Lakes [1] from conventional open cell polymer foams by constructing a re-entrant type of network. Experimental investigations were performed by Choi and Lakes [2,3] for polymer and metallic foams, respectively, with negative Poisson’s ratio.

The initial approaches for the modeling of materials with negative Poisson’s ratio, auxetic materials, were based on the analysis of reticulated structures whose elements are made of beams. See for example [4,5,6,7] where the effects of orientation, cross section, length and elastic properties of the beams assemblage were investigated. Composite materials with Poisson’s ratios close to −1 were analyzed by Milton [8], who studied also a multiscale laminate configuration. A recent attempt to achieve a combination of auxetic behavior and enhanced mechanical properties was made by Assidi and Ganghoffer [9], who suggested to embed auxetic inclusions in a non-auxetic matrix. While most of the investigations on auxetic material were concerned with their elastic properties, the inelastic effects were very rarely considered. To the best of our knowledge, these effects were taken into account only by Overaker *et al.* [10], Deshpande and Fleck [11], and recently by Dirrenberger *et al*. [12,13].

Lee *et al*. [14] were among the first to use the homogenization technique in conjunction with the finite element analysis to predict the effective elastic properties of this type of materials. Some more works employing the numerical homogenization approach are mentioned in the review by Prawoto [15]. Recently, it was shown by Azoti *et al*. [16] that micromechanical models based on Eshelby’s inclusion concept could not capture the overall auxetic behavior of composites made of non-auxetic constituents. This was the case even in composites possessing a re-entrant microstructure, due to the lack of possibility to introduce joints between inclusions.

A micromechanical model based on the homogenization procedure for periodic multiphase materials was developed by Aboudi *et al*. [17,18]. It is capable of predicting the effective thermoelastic moduli, the effect of the inelastic behavior of the constituent phases, damage and other effects. The reliability and accuracy of the moduli and the inelastic response obtained by this approach have been verified by performing extensive comparisons with analytical and finite element solutions under various circumstances (e.g., composites under normal, axial shear and thermal loadings) in the presence and absence of inelastic effects. Moreover, it was shown capable of predicting the properties and behavior of lattice materials [19].

This approach is employed here to predict, in particular, the effective negative Poisson’s ratio of arrays that are capable of generating such values. Porous re-entrant arrays made of non-auxetic material, as well as microstructural two-phase composite arrays, which may provide enhanced mechanical properties, are investigated. It is noted that the presently used approach can be employed in the framework of multi-scale analysis, to predict the characteristics of the architectured inclusion material as well as the macroscopic properties of the type of composites considered in [9]. In addition, material designing process is suggested through a back-out procedure that is employed to determine the (non-auxetic) constituents’ material properties that generate a desired value of the negative effective Poisson’s ratio. The combined effects of a re-entrant array that generates a negative effective Poisson’s ratio and the elastoplastic behavior of its constituents are studied here by a comparison with the response of the same array when it generates a positive Poisson’s ratio.

## 2. Micromechanical Analysis

**Figure 1:**
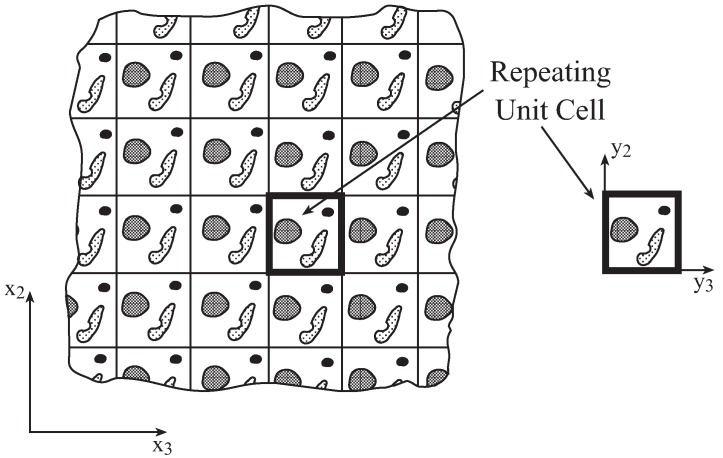
A multiphase composite with a periodic microstructure in the global system $x_{2}\;-\;x_{3}$, characterized by a repeating unit cell (highlighted). The repeating unit cell is given with respect to the local coordinate system $y_{2}-y_{3}$.

The micromechanical model employed to predict the effective thermoelastic properties and inelastic response of multiphase composites is referred to as the high-fidelity generalized method of cells (HFGMC) and is fully described in [17,18]. This model is based on a homogenization technique of composites with periodic microstructure as shown in Figure 1. The repeating unit cell of such a composite is divided into arbitrary number of rectangular subcells, labeled by the indices (β,γ), each of which may contain a distinct homogeneous material. The dimensions of the subcell along the 2 and 3 axes are denoted by $h_{\beta }$ and $l_{\gamma }$, respectively. In the two-dimensional case of continuous fibers, a local coordinate system $\left({\bar{y}}_{2}^{\left(\beta \right)},{\bar{y}}_{3}^{\left(\gamma \right)}\right)$ is introduced in each subcell whose origin is located at its center.

The local (subcell) constitutive equation of the material which, in general, is assumed to be elastoplatic, is given by

(1) $$\sigma^{\left(\beta \gamma \right)}=C^{\left(\beta \gamma \right)}\left(\epsilon^{\left(\beta \gamma \right)}-\epsilon^{I(\beta \gamma )}\right)$$

where $\sigma^{\left(\beta \gamma \right)}$ is the stress tensor in subcell (β,γ), $C^{\left(\beta \gamma \right)}$ is the stiffness tensor of the material in the subcell and $\epsilon^{\left(\beta \gamma \right)},\epsilon^{I(\beta \gamma )}$ are the total and inelastic, respectively. The inelastic strain increments at the various locations within the subcell are calculated using the Prandtl–Reuss flow rule for elastoplastic materials (or an appropriate evolutionary law for viscoplastic materials).

The basic assumption in HFGMC is that the displacement vector in each subcell is given by the quadratic form

(2) $$\begin{array}{ccc} u^{\left(\beta \gamma \right)} & = & \bar{\epsilon }\cdot x+W_{\left(00\right)}^{\left(\beta \gamma \right)}+{\bar{y}}_{2}^{\left(\beta \right)}W_{\left(10\right)}^{\left(\beta \gamma \right)}+{\bar{y}}_{3}^{\left(\gamma \right)}W_{\left(01\right)}^{\left(\beta \gamma \right)}\\ & + & \frac{1}{2}\left(3{\bar{y}}_{2}^{(\beta )2}-\frac{h_{\beta }^{2}}{4}\right)W_{\left(20\right)}^{\left(\beta \gamma \right)}+\frac{1}{2}\left(3{\bar{y}}_{3}^{(\gamma )2}-\frac{l_{\gamma }^{2}}{4}\right)W_{\left(02\right)}^{\left(\beta \gamma \right)} \end{array}$$

where $\bar{\epsilon }$ is the applied (external) strain and the unknown terms $W_{\left(mn\right)}^{\left(\beta \gamma \right)}$ must be determined from the fulfillment of the equilibrium equations, the periodic boundary conditions and the interfacial continuity conditions of displacements and tractions between subcells. A principal ingredient in the present micromechanical analysis is that all these conditions are imposed in the average (integral) sense.

As a result of the imposition of these conditions, a linear system of algebraic equations is obtained, which can be represented in the following form

(3) KU=f+g

where the matrix K contains information on the subcell material properties and dimensions, U contains the unknown terms $W_{\left(mn\right)}^{\left(\beta \gamma \right)}$ in the displacement expansion, Equation (2), the f vector contains information on the externally applied strain and thermal effects, and g contains the inelastic effects expressed by integrals of inelastic strains.

Once Equation (3) is solved. the local stress and strain fields throughout the repeating unit cell can be determined. As a result, the micromechanically established constitutive equations that govern the overall (global) behavior of the multiphase material can be represented in the form

(4) $$\bar{\sigma }=C^{*}\;\left(\bar{\epsilon }-{\bar{\epsilon }}^{I}\right)$$

In this equation, $\bar{\sigma }$ is the average stress in the composite, $C^{*}$ is its effective elastic stiffness tensor, and $\bar{\epsilon },{\bar{\epsilon }}^{I}$ are the overall total and inelastic, respectively. A notable feature of the present model is that it provides closed-form expressions for $C^{*}$ and ${\bar{\epsilon }}^{I}$ in terms of the geometry of the repeating unit cell and the material properties of its constituents. These closed-form expressions have been summarized and presented in [17,18].

## 3. Results and Discussion

The briefly described micromechanical analysis is implemented herein to predict the effective behavior of composites possessing specific microstructural architectures that are capable to generate negative effective Poisson’s ratios and thermal expansion coefficients.

### 3.1. Elasticity Effects

#### 3.1.1. Parametric Study

**Figure 2:**
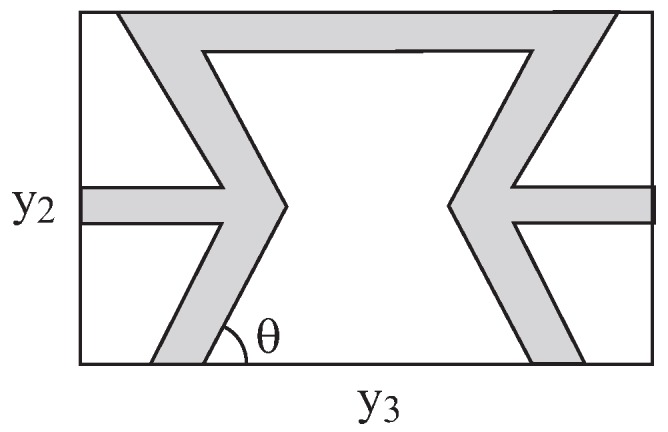
Repeating re-entrant unit cell with angle *θ*. The shaded and white regions are labeled by 1 and 2, respectively.

**Figure 3:**
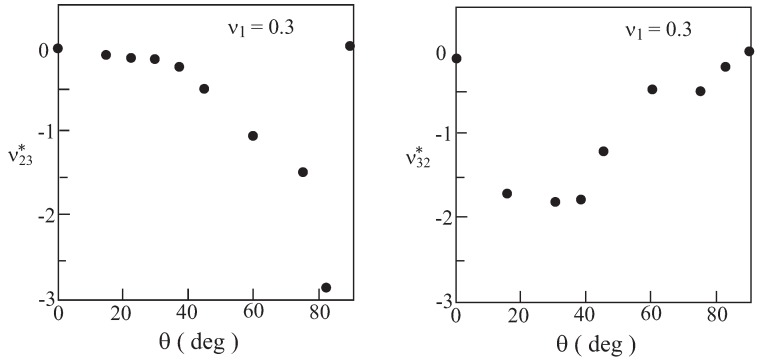
Variation of the effective Poisson ratios $\nu_{23}^{*}$ and $\nu_{32}^{*}$ with *θ* for a material with voids ($v_{f}=0.28$).

For the generation of negative effective Poisson’s ratios, re-entrant microstructural architectures with various values of the acute angles have been constructed. Such forms of re-entrant arrays have been reported by several investigators, see [20,21] for example. In Figure 2, a repeating unit cell is shown. For a material with voids, the shaded area shown in Figure 2 is filled with a solid constituent (labeled by 1) while the rest of the area is kept empty. For all values of *θ*, the volume fraction $v_{f}$ of the solid (shaded) material is kept approximately constant: $v_{f}=0.28$ (*i.e.*, the solid occupies 28% of the total area of the repeating unit cell). It is noted that the re-entrant microstructure yields macroscopic anisotropy, namely different values of effective Poisson ratios $\nu_{23}^{*}$ and $\nu_{32}^{*}$. These effective Poisson’s ratios of the porous microstructured material (made of an elastic isotropic material whose Poisson’s ratio $\nu_{1}$ is equal to 0.3) are shown in Figure 3 against the angle *θ*. This figure clearly exhibits the resulting negative values of Poisson’s ratios as predicted by the micromechanical model. It is noted that there is a steep decrease of $\nu_{23}^{*}$ and a steep increase of $\nu_{32}^{*}$ for $\theta >40^{\circ }$. The configuration with $\theta =90^{\circ }$ yields Poisson’s ratios of a small positive value. This configuration forms a transitional case that separates between re-entrant configurations (for which $\nu_{23}^{*}$ and $\nu_{32}^{*}$ are negative) and honeycomb ones (for which $\nu_{23}^{*}$ and $\nu_{32}^{*}$ are positive). The steep variation and the transition to positive values is similar to the results presented in [7], which are based on the strength of material approach.

It is worth mentioning that the dependence of the effective Poisson ratios on the Poisson ratio of the constituent material, $\nu_{1}$, has been found to be very weak.

It should be emphasized that the Poisson’s ratios of anisotropic material must fulfill the following inequality (see [22])

(5) $$|\nu_{ij}^{*}|<\sqrt{\frac{E_{i}^{*}}{E_{j}^{*}}}$$

where $E_{i}^{*}$ and $E_{j}^{*}$ are the effective Young’s moduli in the *i* and *j*-directions, respectively. This inequality has been checked and verified in all cases.

**Figure 4:**
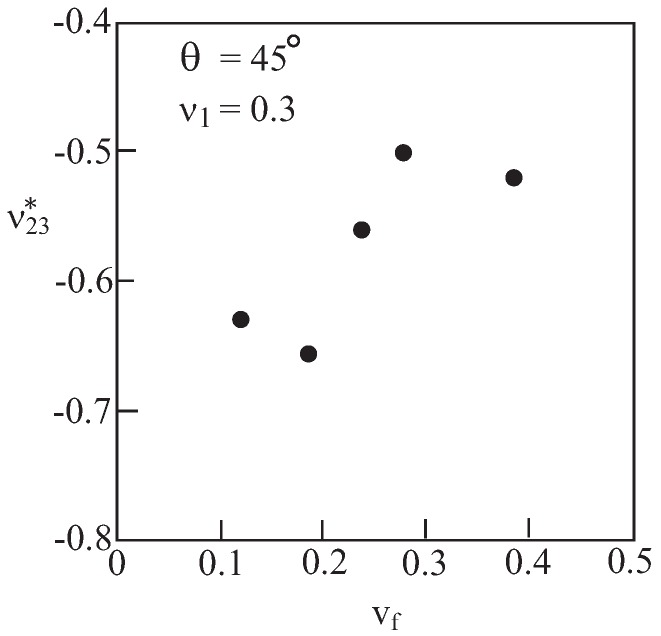
Variation of the effective Poisson’s ratio $\nu_{23}^{*}$ with volume ratio $v_{f}$ of the material with voids ($\theta =45^{\circ }$).

In order to examine the effect of volume fraction variation on the predicted effective negative values of $\nu_{23}^{*}$, five repeating unit cells were considered, in all of which the angle *θ* has been kept constant at $45^{\circ }$ while the volume fraction $v_{f}$ of the material ($\nu_{1}=0.3$) with voids changes. The resulting micromechanical predictions of the effective Poisson’s ratio are shown in Figure 4. It can be concluded that at the present range of values of volume fractions, a weak and not necessarily monotonic dependence on $v_{f}$ is observed.

**Figure 5:**
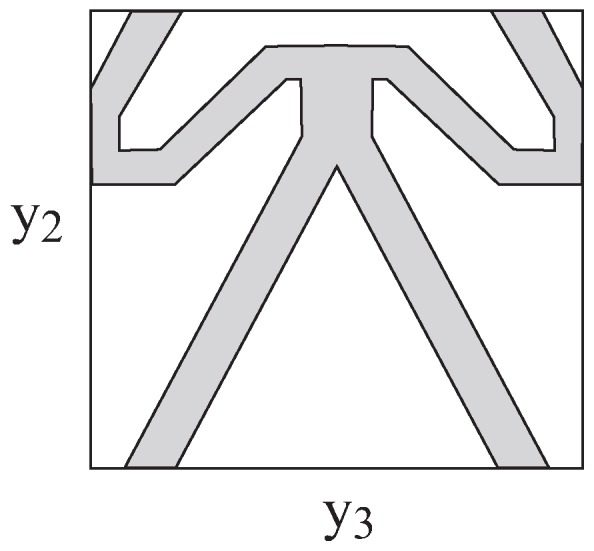
A repeating unit cell is shown for an array that generates negative effective Poisson’s ratios.

The re-entrant array shown in Figure 2 is not the only possible configuration generating negative effective Poisson’s ratios. Another array that is capable to generate negative effective Poisson’s ratios has been designed and fabricated by Xu [21], Larsen *et al*. [23], and discussed by Bendsøe and Sigmund [24]. The repeating unit cell of this array is shown in Figure 5. The shaded region is filled with an elastic material whose volume fraction is $v_{f}=0.28$, while the second region is empty. The implementation of the micromechanical analysis in this case readily predicts that the negative effective Poisson’s ratio of this array is given by $\nu_{23}^{*}=-1.1$.

**Figure 6:**
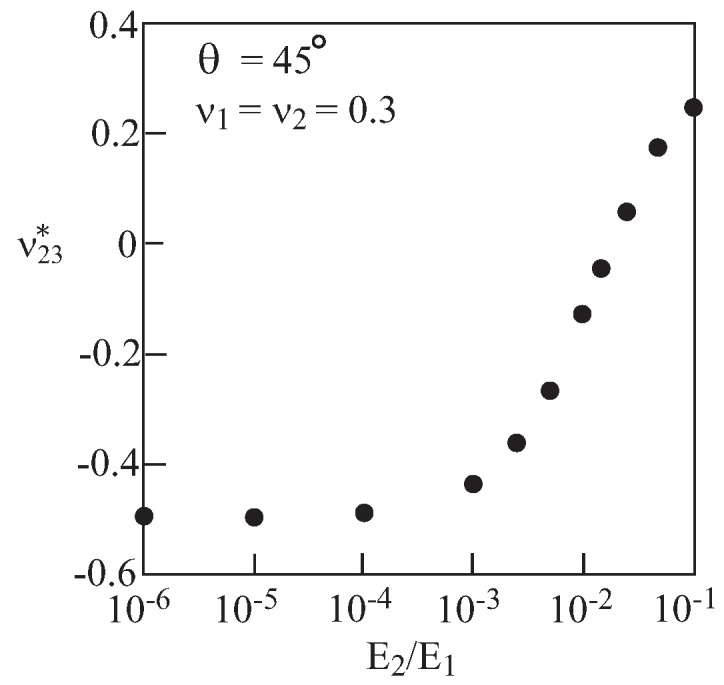
Re-entrant array ($\theta =45^{\circ }$) with two distinct materials. Variation of the effective Poisson’s ratio $\nu_{23}^{*}$ with the Young’s modulus of material 2 while keeping its Poisson’s ratio constant.

**Figure 7:**
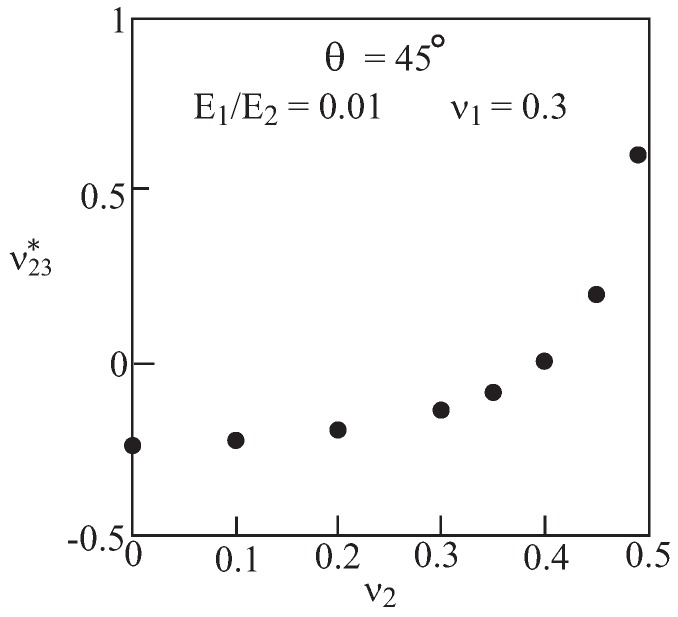
Re-entrant array ($\theta =45^{\circ }$) with two distinct materials. Variation of the effective Poisson’s ratio $\nu_{23}^{*}$ with the Poisson’s ratio of material 2 while keeping its Young’s modulus constant.

So far, effective Poisson’s ratio of materials with voids has been predicted. Thinking of enhanced mechanical properties, it might be useful to consider non-porous materials possessing negative Poisson’s ratios. These may be obtained by filling the voids in the previous configurations with isotropic materials (labeled by 2) with finite (*i.e.*, not zero) properties. To this end, the present micromechanical model, which is capable of predicting the effective Poisson’s ratio of a composite with two (or more) phases, is employed. Figure 6 exhibits the predicted $\nu_{23}^{*}$ for a re-entrant array with $\theta =45^{\circ }$ in which the Young’s modulus $E_{2}$ of material 2 is varied while keeping its Poisson’s ratio $\nu_{2}$ constant. This figure clearly exhibits the transition of $\nu_{23}^{*}$ from negative to positive values as $E_{2}$ increases. Figure 7 exhibits the transition of $\nu_{23}^{*}$ from negative to positive values as the Poisson’s ratio $\nu_{2}$ of material 2 changes while its Young’s modulus $E_{2}$ is kept constant. The results of Figure 6 and Figure 7 offer the possibility of controlling the value of the effective negative Poisson’s ratio without modifying the microstructural architecture. It can be concluded that the micromechanical model provides a tool to enable this design possibility.

#### 3.1.2. Material Design Strategy

For a re-entrant array with given elastic properties $E_{1}$, $\nu_{1}$ of material 1, it is possible to employ the present micromechanical model in order to determine the properties $E_{2}$ and $\nu_{2}$ of the elastic isotropic material that fills region 2 in Figure 2 that provides a composite with a predetermined value of an effective negative Poisson’s ratio $\nu_{23}^{*}$. This design task can be achieved by demanding that the following minimization should be satisfied

(6) $$|\nu_{23\;req}^{*}-\nu_{23\;opt}^{*}|=min$$

where $\nu_{23\;req}^{*}$ is the predetermined Poisson’s ratio that the composite has to provide and $\nu_{23\;opt}^{*}$ is the closest value obtained by the optimization procedure. The latter is performed by a commercial code using the sequential linear programming method.

Table 1 shows the properties of material 2 and the effective transverse Young’s moduli of the re-entrant array with $\theta =45^{\circ }$ for various values of $\nu_{23\;req}^{*}$, which are practically equal to $\nu_{23\;opt}^{*}$. As it is expected, $\nu_{23}^{*}=-0.5$ is the lowest value that can be obtained from the present array.

**Table 1:** For a required negative value of $\nu_{23}^{*}$, optimal material 2 properties in re-entrant array $\theta =45^{\circ }$ of Figure 2 and the resulting effective transverse moduli.

$\nu_{23}^{*}$	$E_{2}/E_{1}$	$\nu_{2}$	$E_{2}^{*}/E_{1}$	$E_{3}^{*}/E_{1}$
−0.1	$0.54\times 10^{-2}$	0.3	$0.32\times 10^{-1}$	$0.76\times 10^{-1}$
−0.2	$0.30\times 10^{-2}$	0.3	$0.21\times 10^{-1}$	$0.58\times 10^{-1}$
−0.3	$0.15\times 10^{-2}$	0.3	$0.13\times 10^{-1}$	$0.40\times 10^{-1}$
−0.4	$0.61\times 10^{-3}$	0.3	$0.57\times 10^{-2}$	$0.20\times 10^{-1}$
−0.5	0	–	0	0

### 3.2. Plasticity Effects

**Figure 8:**
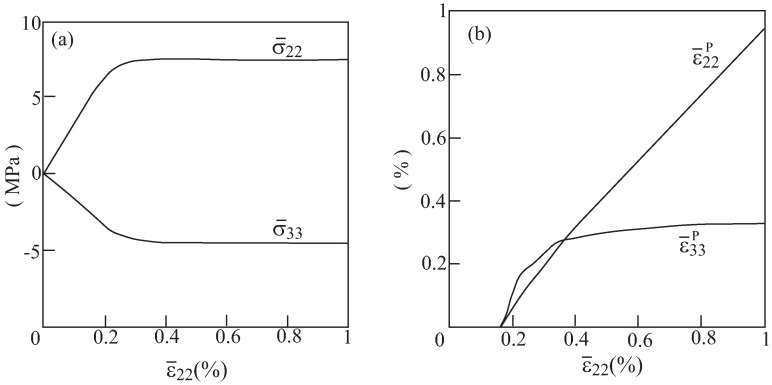
The elastoplastic response of aluminum with voids in the re-entrant array with $\theta =75^{\circ }$ that generates a negative effective elastic Poisson’s ratio. (**a**) Average transverse stresses and (**b**) transverse plastic strains.

**Figure 9:**
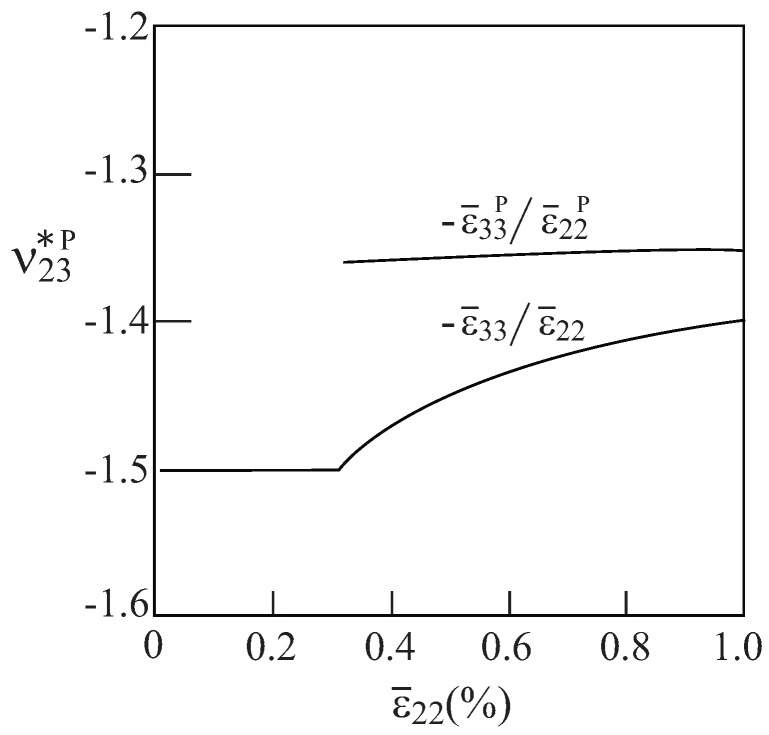
Plastic and apparent Poisson ratio of aluminum with voids in the re-entrant array with $\theta =75^{\circ }$, under uniaxial stress in the $y_{2}$ direction.

It should be interesting to investigate the mutual effects of a negative Poisson’s ratio $\nu_{23}^{*}$ and the elastoplastic behavior of a re-entrant array with voids. To this end, let us assume that region 1 (shaded) in Figure 2 of an array with $\theta =75^{\circ }$ are filled with an aluminum alloy, which is assumed (for simplicity) to behave as an elastic perfectly-plastic material whose Young’s modulus, Poisson’s ratio and yield stress are given by $E_{1}=55.15$ GPa, $\nu_{1}=0.3$ and $Y_{1}=90$ MPa, respectively. As was shown in Figure 3, this array generates in the elastic region an effective Poisson’s ratio $\nu_{23}^{*}=-1.5$. The response of the composite under uniaxial strain loading (in which ${\bar{\epsilon }}_{22}\neq 0$) is investigated. The resulting overall behavior is shown in Figure 8. This figure shows the two average transverse stresses ${\bar{\sigma }}_{22}$, ${\bar{\sigma }}_{33}$ which have different signs, and the corresponding plastic strains ${\bar{\epsilon }}_{22}^{P}$, ${\bar{\epsilon }}_{33}^{P}$ having the same sign. This indicates that the evolution of plasticity does not eliminate the auxetic nature of the material as it has already been demonstrated in Dirrenberger *et al*. [13] for chiral arrays. The issue of defining the Poisson’s ratio in the presence of plasticity has been addressed by Deshpande and Fleck [11] and Dirrenberger *et al*. [13] who presented the plastic and the apparent Poisson ratio, respectively. These two parameters are shown in Figure 9 for the above-defined configuration subjected to uniaxial stress loading (in which ${\bar{\sigma }}_{22}\neq 0$). The variation of the plastic Poisson ratio, ${\bar{\epsilon }}_{33}^{p}/{\bar{\epsilon }}_{22}^{p}$, and the apparent Poisson ratio, ${\bar{\epsilon }}_{33}/{\bar{\epsilon }}_{22}$, with the applied load clearly indicates that plasticity becomes significant when ${\bar{\epsilon }}_{22}$ exceeds 0.003. While the plastic Poisson ratio remains almost constant, the absolute value of the apparent Poisson ratio decreases with the accumulation of plastic strains. However, both parameters remain negative in the presence of plasticity.

**Figure 10:**
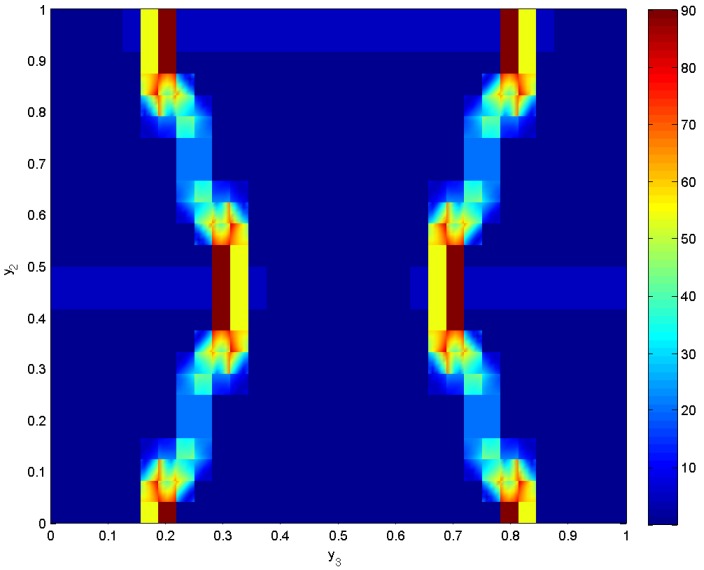
The von Misses stress distribution over the re-entrant repeating unit cell of aluminum with voids with $\theta =75^{\circ }$.

The stress concentration under the above studied state of uniaxial stress is presented in Figure 10, which shows the von Misses stress. The observation that stresses of high magnitude, up to 90 MPa, develop near the “joints" is in accordance with results presented by Dirrenberger *et al*. [13] in chiral arrays.

**Figure 11:**
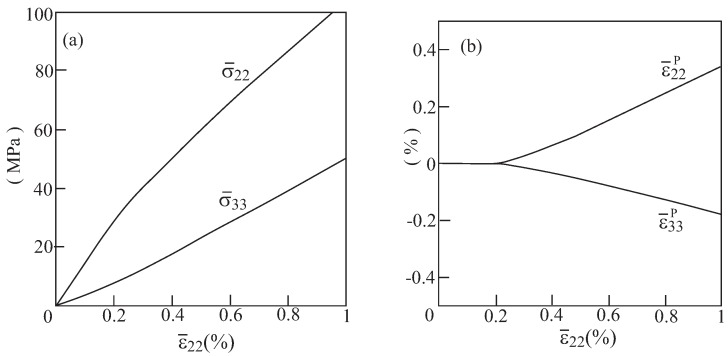
The elastoplastic response of aluminum and elastic material in the re-entrant array with $\theta =75^{\circ }$ that generates a positive effective elastic Poisson’s ratio. (**a**) Average transverse stresses and (**b**) transverse plastic strains.

In order to further assess the effect of the negative elastic Poisson’s ratio of the studied re-entrant array, let the region of material 2 in this array be filled with elastic isotropic material such that $E_{2}/E_{1}=0.1$ and $\nu_{2}=0.3$. This array generates, in the elastic domain, a positive Poisson’s ratio: $\nu_{23}^{*}=0.22$. The elastoplastic response of this array to uniaxial strain is shown in Figure 11. It can be immediately observed that the transverse stresses have the same sign, but the plastic strains differ in sign. Both these latter results are known to occur in standard elastoplastic composites (with $\nu_{23}^{*}>0$).

**Figure 12:**
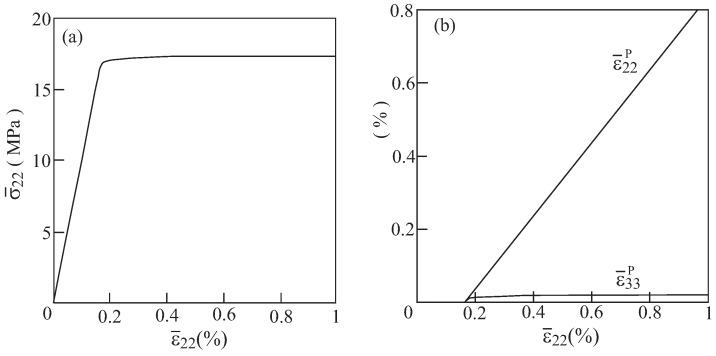
The elastoplastic response of aluminum with voids in the array with $\theta =90^{\circ }$ that generates a positive effective elastic Poisson’s ratio. (**a**) Average transverse stress and (**b**) transverse plastic strains.

Another array of elastoplastic material with voids that generates positive $\nu_{23}^{*}$ in the elastic domain is the one with $\theta =90^{\circ }$ that predicts that $\nu_{23}^{*}=0.013$. The response of the elastic perfectly-plastic aluminum of this array is shown in Figure 12. Here ${\bar{\sigma }}_{33}$ is negligibly small and ${\bar{\epsilon }}_{33}^{P}$ is positive. This behavior does not correspond to the elastoplastic response of standard composite with positive effective Poisson’s ratio and can be attributed to the fact that the present configuration is, as stated before, a transitional one with very small $\nu_{23}^{*}$.

## 4. Conclusions

A continuum-based micromechanical approach has been employed for the analysis of periodic microstructural arrays possessing negative Poisson’s ratios. Elastic effective properties as well as inelastic behavior were obtained. In addition to a porous material, a configuration of non-porous two-phase composite possessing negative Poisson’s ratios, obtained by filling the voids of the re-entrant configuration with a second isotropic material, has been considered. Its constituents’ properties for a required negative value of Poisson’s ratio have been deduced by a back-out application of the micromechanical model.

The present approach, which has been used here to predict also the inelastic behavior of the microstructural array, can be extended to include other effects such as viscoelasticity, damage and large deformations. Moreover, the presently applied approach was recently modified by Haj Ali and Aboudi [25] to enable efficient handling of various microstructural architectures. It is a future work plan to employ this strategy for the analysis of the behavior of auxetic material of both ordered and disordered structure.
